# Short‐term outcomes of open liver resection and laparoscopic liver resection: Secondary analysis of data from a multicenter prospective study (CSGO‐HBP‐004)

**DOI:** 10.1002/ags3.12046

**Published:** 2017-10-23

**Authors:** Shogo Kobayashi, Keisuke Fukui, Yutaka Takeda, Shin Nakahira, Masanori Tsujie, Junzo Shimizu, Atsushi Miyamoto, Hidetoshi Eguchi, Hiroaki Nagano, Yuichiro Doki, Masaki Mori

**Affiliations:** ^1^ Department of Surgery Osaka Medical Center for Cancer and Cardiovascular Diseases Osaka Japan; ^2^ Department of Surgery Osaka University Hospital Osaka Japan; ^3^ Department of Surgery Osaka International Cancer Institute Osaka Japan; ^4^ Osaka Medical Center for Cancer and Cardiovascular Diseases Center for Cancer Control and Statistics Osaka Japan; ^5^ Osaka International Cancer Institute Center for Cancer Control and Statistics Osaka Japan; ^6^ Department of Surgery Kansai Rosai Hospital Amagasaki Japan; ^7^ Department of Surgery Nara Hospital Kinki University Faculty of Medicine Ikoma Japan; ^8^ Department of Surgery Osaka Rosai Hospital Sakai Japan; ^9^ Department of Hepatobiliary‐Pancreatic Surgery National Hospital Organization Osaka National Hospital Osaka Japan

**Keywords:** laparoscopy, liver, morbidity

## Abstract

The aim of the present study was to compare short‐term outcomes of laparoscopic and open liver resection (LLR and OLR, respectively), and we first analyzed a preoperatively enrolled and prospectively collected database. We carried out a secondary analysis using a preoperative enrolled database that included the details of 786 patients who had been enrolled in a previously carried out randomized controlled trial to assess short‐term outcomes, including morbidities. Statistical analyses included logistic regression, propensity score matching (PSM) with replacement, and inverse probability of treatment weighting (IPTW) analyses. Among 780 liver resections, OLR was carried out in 543 patients and LLR was carried out in 237 patients. LLR was selected in patients with a worse liver function and was related to a smaller resected liver weight and/or partial resection. Logistic regression, PSM, and IPTW analyses revealed that LLR was associated with less blood loss and a lower incidence of morbidities, but a longer operating time. LLR was found to be a preferred factor in biliary leakage by IPTW only. LLR was a preferred factor for blood loss, morbidities and hospital stay, but was associated with a longer operating time. UMIN‐CTR, UMIN000003324.

## 1 INTRODUCTION

Laparoscopic surgical techniques have recently been applied to liver resection,^1^, ^2^, ^3^, ^4^, ^5^ despite the fact that their feasibility remains controversial. Although several randomized controlled trials (RCT) have been carried out to investigate the usefulness of laparoscopic techniques in gastric and colorectal surgery,^6^, ^7^, ^8^ laparoscopic liver resection (LLR) has a relatively short history and the surgical techniques are still under development. As a consequence, it remains difficult to control quality in RCT. Thus, most studies on LLR and open liver resection (OLR) have been retrospective in nature and have analyzed a relatively small number of patients at a single hospital,^9^, ^10^, ^11^ whereas other studies have used a nationwide database of postoperatively enrolled patients.^12^, ^13^, ^14^ Thus, the potential for selection bias, enrolment bias, and missing values could not be avoided.

We previously carried out a multicenter RCT in which the endpoint was short‐term surgical outcome.^15^ Patients in the database were classified according to the method that was used to seal the liver cut surface. Briefly, over 700 patients from 11 institutes were enrolled in the RCT. Results showed that incidence of postoperative bile leakage and bleeding among the methods of sealing did not differ to a statistically significant extent. LLR accounted for approximately one‐third of the procedures that were included in the database. All of the patients were enrolled preoperatively. Perioperative factors that were recorded in the database included: liver function, hepatitis, type of resection, operating time, blood loss, resected liver weight, detailed morbidities, and hospital stay.

We are of the opinion that the database would be useful for analysis of short‐term outcomes of patients undergoing LLR and OLR for several reasons: patients were enrolled preoperatively, short‐term surgical outcome and all morbidities were collected prospectively, and data were obtained from a multi‐institutional study and therefore showed universality. Although an RCT is necessary to make a precise comparison, the results of the analyses in the present study would provide a useful rationale for the carrying out an RCT.

The present study shows how patients were selected for LLR and compares short‐term outcomes between OLR and LLR. Propensity score matching (PSM) and inverse probability of treatment weighting (IPTW) analyses were used to reduce selection bias. Results showed that LLR was associated with less blood loss and a lower incidence of morbidities, but a prolonged operating time. These results provide information that can be used until completion of an RCT. The results are useful for determining the indications for LLR and for obtaining informed consent from patients in whom LLR is indicated.

## 2 METHODS

### 2.1 Study design

In the present study, we aimed to carry out a secondary analysis of the data obtained in a previous open, multicenter RCT that was carried out to explore the efficacy of fibrin sealant (FS) with polyglycolic acid (PGA) versus fibrinogen‐based collagen fleece (CF) in preventing postoperative biliary leakage and/or hemorrhage at the liver cut surface.^15^ The trial was started by the Clinical Study Group of Osaka University (CSGO), the Hepato‐Biliary‐Pancreatic (HBP) Group from November 2009 to May 2014. Review boards of each institution approved the protocol, and written informed consent was obtained from each patient.

A total of 786 patients from 11 institutions were enrolled and randomly assigned to the PGA‐FS group (n = 391) or to the CF (n = 395) group. The following data were collected: age, gender, body height, bodyweight, preoperative platelet count, biochemical data, prothrombin time, hepatitis virus status, primary diseases, type of resection (laparoscopic or open), type of hepatectomy (partial, segmentectomy, sectionectomy etc.), liver resection weight, operative time, estimated blood loss, postoperative morbidities (bile leakage, hemorrhage, and other morbidities), and postoperative hospital stay.

As a result of the lack of an international definition of biliary leakage^16^ when planning this RCT, we defined biliary leakage as a drain bilirubin to serum bilirubin ratio of ≥5. When the ratio was 3 to <5, we re‐measured the drain and serum bilirubin levels after 2 or 3 days. Postoperative hemorrhage was defined by the need for re‐laparotomy or transfusion to achieve hemostasis.

Patient selection was carried out as follows. Patients in whom hepatectomy was planned and who were ≥20 years of age were enrolled in the RCT and preoperatively assigned. Type of resection (laparoscopic or open resection and presence or absence of biliary reconstruction) and reason for hepatectomy were not restricted.

Hepatectomy was carried out according to each institution's method with board‐certified expert surgeons or instructors (hepatobiliary‐pancreatic field) of the Japanese Society of Hepato‐Biliary‐Pancreatic Surgery. Each surgeon decided whether a bubble leakage test should be done. After achieving primary hemostasis by suturing or electrocautery and after suturing of any sites with obvious biliary leakage, PGA‐FS or CF was applied to the cut surface of the liver to avoid the possibility of FS breakdown as a result of bile contamination.^17^ Each surgeon determined whether or not to leave a drainage tube in the liver cut surface to gain information about postoperative bile leakage and hemorrhage. Procedures were confirmed at a CSGO meeting every 3 months.

### 2.2 Statistical analysis

**Figure 1:**
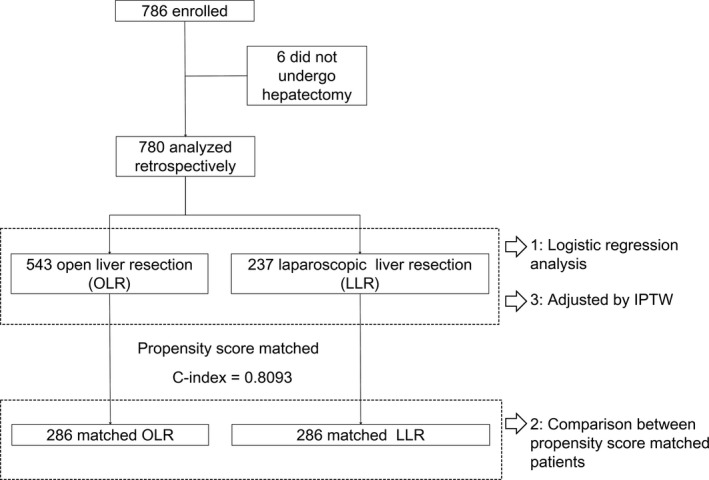
Flow diagram of the present study. IPTW, inverse probability of treatment weighted; LLR, laparoscopic liver resection; OLR, open liver resection

Statistical analyses were carried out according to the flow diagram in Figure 1. Methods of the analyses are described below.

The data were expressed as the mean. Differences between groups were tested using Student's *t* test or the chi‐squared test, as appropriate. *P* values of <.05 were considered to indicate statistical significance. A multivariate logistic regression analysis was performed. PSM analysis was performed with replacement to increase the average quality of matching and to decrease bias.^18^, ^19^ IPTW was used to adjust for differences and reduce the impact of any treatment selection bias.^20^ With this method, the weights for patients who were treated with LLR were the inverse of the propensity score (determined by logistic regression); the weights for patients who underwent OLR were the inverse of the 1‐propensity score. Logistic regression was used to estimate the propensity scores. The following variables were included in the model: type of resection, type of resection, age, gender, platelet, bilirubin, albumin, prothrombin time, HBs‐antigen positivity and HCV‐antibody positivity. To visualize hazard ratio of surgical outcomes and similarity between analyses, we used a forest plot. All of the statistical analyses were conducted using the R software program (version 2.15.2, Foundation for Statistical Computing, Vienna, Austria, http://www.r-project.org).

## 3 RESULTS

### 3.1 Patient flow

A total of 786 patients were enrolled in the present study. Among these patients, 780 patients underwent hepatectomy and were analyzed in the present study (Figure 1). OLR was carried out in 543 patients and LLR was carried out in 237 patients. We first carried out a logistic regression analysis using all of the patient data. Next, we carried out a PSM analysis after confirming that the C‐index was >.8 (C‐index: .8093). PSM analysis was done with replacement to increase the average quality of matching and to decrease bias.^18^, ^19^ Two hundred and eighty‐six patients from each group were included in the PSM analysis by replacement. Finally, we carried out an IPTW analysis to reduce selection bias and to analyze all of the data in the database.

### 3.2 Demographic characteristics of the patients who underwent OLR and LLR

We summarized the demographic characteristics of the patients who underwent OLR and LLR (Table 1 **)**. In the trial database, platelet count and prothrombin time of the LLR group was lower, whereas the rate of HCV positivity was higher in comparison to the OLR group. Regarding type of liver resection, the rate of partial resection was higher in the LLR group. Liver resection weight, estimated blood loss, and duration of postoperative hospital stay in the LLR group were lower in comparison to the OLR group. Rates of biliary leakage and other postoperative adverse events were lower in the LLR group.

**Table 1:** Comparison of demographic characteristics of enrolled patients between OLR and LLR

	OLR (n = 543)	LLR (n = 237)	*P*‐value
Age (years)	64.78 ± 12.82	65.55 ± 13.78	.450
Gender			
Male/Female	363/178 (66.9%/32.8%)	151/85 (63.7%/35.9%)	.446
Platelets (×10^4^/μL)	19.94 ± 7.91	17.9 ± 7.85	.001
Creatinine (mg/dL)	0.79 ± 0.33	0.85 ± 0.60	.101
Total bilirubin (mg/dL)	0.69 ± 0.37	0.70 ± 0.32	.809
Albumin (g/dL)	3.95 ± 0.46	3.97 ± 0.44	.602
Prothrombin time (%)	91.41 ± 15.53	86.63 ± 15.97	<.001
HBsAg‐positive	47 (8.7%)	20 (8.4%)	.998
HCVAb‐positive	115 (21.2%)	76 (32.1%)	.002
Surgical procedure (partial resection)	253 (46.6%)	152 (64.1%)	<.001
Liver resection weight	306.43 ± 340.26	166.57 ± 227.79	<.001
Operating time (min)	329.61 ± 64.71	330.62 ± 180.20	.940
Estimated blood loss (g)	833.43 ± 1334.78	361.12 ± 1118.09	<.001
Biliary leakage in CSGO‐HBP‐004 Study	31 (5.7%)	5 (2.1%)	.044
Biliary leakage in ISGLS	69 (12.7%)	13 (5.5%)	.004
Hospital stay (days)	23.27 ± 22.82	14.1 ± 10.85	<.001
Other adverse events	142 (26.2%)	26 (11.0%)	<.001

Values represent mean ± SD or number (%). CSGO, Clinical Study Group of Osaka University; HBP, Hepato‐Biliary‐Pancreatic Group; HBsAg, hepatitis B virus surface antigen; HCVAb, hepatitis C virus antibody; ISGLS, International Study Group of Liver Surgery; LLR, laparoscopic liver resection; OLR, open liver resection.

In summary, laparoscopic resection was selected in patients with a worse liver function (lower platelet count, lower prothrombin time, and HCV positivity). Laparoscopic operative procedures were related to a smaller liver resection weight and/or partial resection.

### 3.3 Surgical outcomes of OLR and LLR (analyzed by logistic regression, PSM, and IPTW)

We analyzed estimated blood loss, operating time, biliary leakage, other adverse events and duration of postoperative hospital stay as the surgical outcomes of liver resection. Biliary leakage was defined according to the definition of the International Study Group of Liver Surgery (ISGLS).^16^

**Figure 2:**
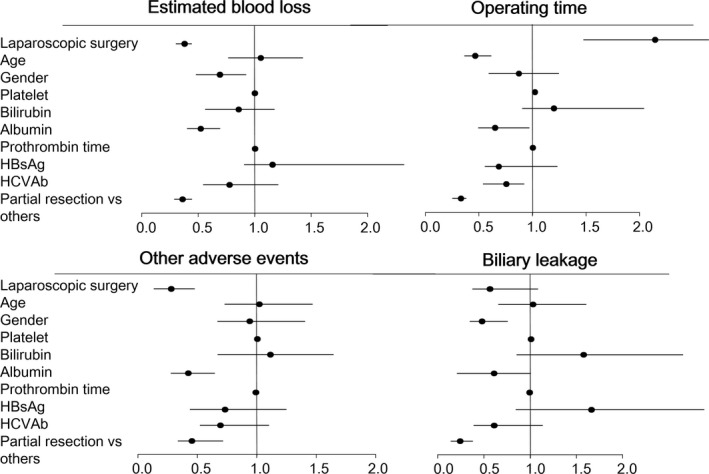
Forest plot of the odds ratios calculated in the logistic regression analysis. HBsAg, hepatitis B virus surface antigen; HCVAb, hepatitis C virus antibody

**Figure 3:**
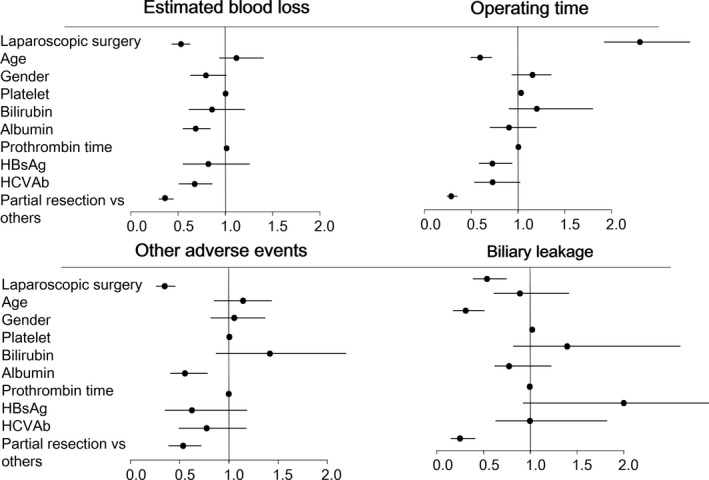
Forest plot of the odds ratios determined in the inverse probability of treatment weighting analysis. HBsAg, hepatitis B virus surface antigen; HCVAb, hepatitis C virus antibody

With regard to blood loss, logistic regression analysis showed that LLR, albumin, and partial resection were associated with less estimated blood loss (Figure 2). After PSM, LLR remained a preferred factor (Table 2). In the IPTW analysis, LLR, albumin, HCV positivity, and partial resection were preferred factors (Figure 3).

**Table 2:** Comparison of the outcomes of propensity score‐matched patients who underwent OLR or LLR

	PS score‐matched OLR (n = 286)	PS score‐matched LLR (n = 286)	*P*‐value
Age (years)	64.63 ± 14.48	64.75 ± 13.87	.918
Gender			
Male/Female	186/100 (65.0%/35.0%)	183/103 (64.0%/36.0%)	.861
Platelets (×10^4^/μL)	17.66 ± 7.52	18.27 ± 8.19	.354
Creatinine (mg/dL)	0.80 ± 0.25	0.83 ± 0.55	.446
Total bilirubin (mg/dL)	0.69 ± 0.27	0.68 ± 0.30	.646
Albumin (g/dL)	3.94 ± 0.46	3.97 ± 0.45	.397
Prothrombin time (%)	89.23 ± 15.50	88.15 ± 15.83	.410
HBsAg‐positive	25 (8.7%)	23 (8.0%)	.880
HCVAb‐positive	76 (26.6%)	79 (27.6%)	.851
Surgical procedure (partial resection)	185 (64.7%)	176 (61.5%)	.488
Liver resection weight	173.55 ± 199.69	172.55 ± 230.99	.956
Operating time (min)	273.65 ± 117.43	335.71 ± 166.71	<.001
Estimated blood loss (g)	404.73 ± 529.94	298.91 ± 552.02	.020
Biliary leakage on CSGO‐HBP‐004 Study	5 (1.7%)	6 (2.1%)	1.000
Biliary leakage on ISGLS	16 (5.6%)	15 (5.2%)	1.000
Other adverse events	49 (17.1%)	24 (8.4%)	.003
Hospital stay (days)	17.09 ± 13.63	13.98 ± 10.51	.002

Values represent mean ± SD or number (%). CSGO, Clinical Study Group of Osaka University; HBP, Hepato‐Biliary‐Pancreatic Group; HBsAg, hepatitis B virus surface antigen; HCVAb, hepatitis C virus antibody; ISGLS, International Study Group of Liver Surgery; LLR, laparoscopic liver resection; OLR, open liver resection.

With regard to operative time, logistic regression analysis showed that age, albumin, HCV positivity, and partial resection were associated with a shorter operative time, whereas LLR was associated with a longer operative time (Figure 2). After PSM, LLR was still associated with a longer operative time (Table 2). In IPTW analysis, age, albumin, HBs‐positivity, and partial resection were preferred factors, whereas LLR remained associated with a longer operative time (Figure 3).

Different factors were found to be associated with biliary leakage and other postoperative adverse events. With regard to postoperative adverse events other than biliary leakage, LLR, albumin, partial resection were preferred factors (Figure 2). After PSM, LLR remained a preferred factor (Table 2). In IPTW analysis, LLR, albumin, and partial resection were preferred factors (Figure 3). Regarding biliary leakage, in logistic regression analysis, male sex and partial resection were preferred factors (Figure 2). After PSM, LLR was not associated with biliary leakage (Table 2). In IPTW analysis, LLR, male sex, and partial resection were preferred factors (Figure 3).

Regarding length of hospital stay, LLR and partial resection were preferred factors. After PSM, LLR remained a preferred factor (Table 2). In IPTW analysis, LLR and partial resection were preferred factors (Figure 3).

In summary, LLR was a preferred factor for estimated blood loss, adverse events and hospital stay. However, the relationship between LLR and biliary leakage depended on the type of analysis. LLR was associated with a longer operative time.

## 4 DISCUSSION

Numerous retrospective studies have investigated the short‐term outcomes of LLR.^21^, ^22^ Some were case–control studies that analyzed more than 200 patients^23^, ^24^; others used PSM to analyze nationwide large‐scale databases.^12^, ^13^, ^14^ However, retrospective studies are associated with certain limitations: they might lack cases and/or information, and they involve selection and enrolment biases. Thus, retrospective studies risk underestimating the incidence of morbidities.

In the present study, we used a preoperatively enrolled and prospectively collected database to investigate perioperative morbidities; a multivariate analysis was carried out. Primary and secondary measures of the database were morbidities.^15^ These data were prospectively collected from almost 800 patients who were preoperatively enrolled; thus, although some bias remains with regard to surgical technique, reliability of this database and the analysis were higher than in any other studies other than RCT. The study was carried out during the transition period between LLR and OLR; both types of resection were well balanced and there were no problems with the analysis. With regard to the quality of the surgical techniques, especially in LLR, our group had a meeting every 3 months, the participating institutions were affiliated with each other, and we always maintained technical cooperation and shared information; thus, any technical bias among the institutions was limited.

Incidence of biliary leakage, defined according to the definition of our previous trial,^15^ was lower (2.1%‐5.7%) in comparison to the incidence when the ISGLS definition was applied (5.5%‐12.7%). Our original definition was more appropriate for the clinical setting and the definition was determined based on clinical data^25^: in cases in which there was no biliary leakage according to our definition, the drainage tube could be removed without any complications. However, the incidence of biliary leakage according to this definition was too low to analyze in the multivariate analysis. We therefore used the definition of the ISGLS in the present study.

Next, we compared the short‐term outcomes observed in the present study with those of previous reports (Table 3).^9^, ^13^, ^14^, ^26^, ^27^, ^28^, ^29^, ^30^, ^31^, ^32^ Almost all of the reports showed less blood loss and a shorter hospital stay; however, there were discrepancies among the reports with regard to operating time and incidence of morbidities. As Table 3 shows, in most of the previous reports, less than 100 patients underwent LLR. One report analyzed over 300 LLR patients^13^ and another analyzed over 900 major LLR patients using PSM;^31^ however, the studies used a nationwide database with postoperative enrolment, and the comparison between our study and previous studies was too difficult. Almost all previous studies suggested that LLR would be associated with less blood loss; however, the other short‐term outcomes were controversial. The possible causes of these differences would be enrolment bias, missing values, surgical bias between hospitals, and selection bias. To reduce enrolment bias, missing values, and surgical bias, we conducted preoperative enrolment and restriction of surgeon and hospital. To reduce selection bias, we need to conduct RCT. At present, three RCT to investigate feasibility are currently ongoing: the ORANGE II PLUS trial (NCT01441856), the OSLO CoMet study (NCT01516710), and our study (CSGO‐HBP‐014, umin‐ctr: UMIN000020234). We need to confirm our opinion by RCT.

**Table 3:** Summary of the results of propensity score analysis of the outcomes of laparoscopic and open liver resection

Ref	Author	Journal	Year	LLR	OLR	Blood loss	Operating time	Morbidity	Hospital stay
26	Cannon et al.	Surgery.	2012	35	140	Less	NSD	Less	Shorter
27	Kim et al.	Surg Endosc.	2014	29	29	NSD	NSD	NSD	Shorter
28	Lin et al.	Int J Colorectal Dis.	2015	36	36	Less	Longer	NSD	Shorter
29	de Angelis et al.	J Laparoendosc Adv Surg Tech A.	2015	52	52	Less	NSD	NSD	Shorter
30	Beppu et al.	Anticancer Res.	2015	52	52	Less	Shorter		Shorter
14	Beppu et al.	J Hepatobiliary Pancreat Sci.	2015	171	342	Less	NSD	NSD	Shorter
13	Takahara et al.	J Hepatobiliary Pancreat Sci.	2015	387	387	Less	Longer	Less	Shorter
31	Takahara et al.	J Hepatobiliary Pancreat Sci.	2016	929	929	Less	Longer	Less	NSD
32	Sposito et al.	Br J Surg.	2016	43	43	NSD	NA	Less	Shorter
9	Cheung et al.	Ann Surg.	2016	110	330	Less	Shorter	NSD	Shorter
	Present study			286	286	Less	Longer	Less	Shorter

Values indicate number of patients. LLR, laparoscopic liver resection; NA, not available; NSD, no statistical difference; OLR, open liver resection.

The present study is associated with some limitations. This study was not randomized and there was a selection bias because the procedure was selected by the surgeon. We did not show superiority of LLR, as LLR was associated with decreased estimated blood loss but an increased operating time. Surgical techniques and devices will continue to be developed and the short‐term outcomes of LLR will change in the future. Another limitation is that we carried out this study as the secondary analysis of a previously carried out study, and we need additional data such as depth of tumor from the liver surface for further analyses. The other limitation is that we could not investigate the prognosis of the patients as the database did not include the prognosis because our former randomized trial investigated the short‐term surgical outcomes and morbidities in patients undergoing liver resection. We would expect LLR to contribute to patient survival because the surgical manipulations carried out in OLR have the potential to lead to surgery‐related recurrence;^33^ however, it would be difficult to detect a difference. Takahara et al.^13^ carried out a PSM analysis and showed that disease‐free survival increased by 10% at 200‐1500 days after resection (LLR, n = 387); however, the difference was not statistically significant. Detection of resection‐related and metastasis‐related factors at the molecular level, such as circulating tumor cells,^33^ would lead to advances in surgical treatment.

Taken together, we analyzed the data from a preoperatively enrolled database to investigate the incidence of perioperative morbidities and showed that LLR was a preferred factor for blood loss and morbidities, but that it was associated with a longer operating time in comparison to OLR.

## DISCLOSURE

The protocol for this research project was approved by an Institutional Reviewer Board and it conforms to the provisions of the Declaration of Helsinki. Committee of Osaka University, School of Medicine, Approval No. 09012. Written informed consent was obtained from each patient. The study was registered at UMIN‐CTR (UMIN000020234, http://www.umin.ac.jp/ctr/index-j.htm).

Conflicts of Interest: Authors declare no conflicts of interest for this article.

Author Contribution: All authors: conception, design, interpretation of data. SK, YT, SN, MT, JS, AM, HE, HN, YD, MM: surgery and acquisition of data. SK and KF: data analysis.

## APPENDIX 1

### CLINICAL STUDY GROUP OF OSAKA UNIVERSITY (CSGO) HEPATO‐BILIARY‐PANCREATIC GROUP

Ikeda Municipal Hospital, Japan Community Health Care Organization Osaka Hospital, Kansai Rosai Hospital, Nara Hospital Kinki University Faculty of Medicine, National Hospital Organization Osaka National Hospital, Osaka Medical Center for Cancer and Cardiovascular Diseases, Osaka Police Hospital, Osaka Rosai Hospital, Osaka University Hospital, Sakai Municipal Hospital, and Suita Municipal Hospital, Osaka Medical Center for Cancer and Cardiovascular Diseases, Osaka International Cancer Institute, Osaka University Hospital, Kansai Rosai Hospital, Nara Hospital Kinki University Faculty of Medicine, Osaka Rosai Hospital, National Hospital Organization, Osaka National Hospital.
